# Genetic deficiency and pharmacological modulation of RORα regulate laser-induced choroidal neovascularization

**DOI:** 10.18632/aging.204480

**Published:** 2023-01-10

**Authors:** Chi-Hsiu Liu, Felix Yemanyi, Kiran Bora, Neetu Kushwah, Alexandra K. Blomfield, Theodore M. Kamenecka, John Paul SanGiovanni, Ye Sun, Laura A. Solt, Jing Chen

**Affiliations:** 1 Department of Ophthalmology, Boston Children’s Hospital, Harvard Medical School, Boston, MA 02115, USA; 2 Department of Molecular Medicine, UF Scripps Biomedical Research, Jupiter, FL 33458, USA; 3 BIO5 Institute and Department of Nutritional Sciences, University of Arizona, Tucson, AZ 85719, USA; 4 Department of Immunology and Microbiology, UF Scripps Biomedical Research, Jupiter, FL 33458, USA

**Keywords:** age-related macular degeneration, angiogenesis, choroidal neovascularization, inflammation, nuclear receptors, RORα, VEGFR2, TNFα

## Abstract

Choroidal neovascularization (CNV) causes acute vision loss in neovascular age-related macular degeneration (AMD). Genetic variations of the nuclear receptor RAR-related orphan receptor alpha (RORα) have been linked with neovascular AMD, yet its specific role in pathological CNV development is not entirely clear. In this study, we showed that *Rora* was highly expressed in the mouse choroid compared with the retina, and genetic loss of RORα in Staggerer mice (*Rorasg/sg*) led to increased expression levels of *Vegfr2* and *Tnfa* in the choroid and retinal pigment epithelium (RPE) complex. In a mouse model of laser-induced CNV, RORα expression was highly increased in the choroidal/RPE complex post-laser, and loss of RORα in *Rorasg/sg* eyes significantly worsened CNV with increased lesion size and vascular leakage, associated with increased levels of VEGFR2 and TNFα proteins. Pharmacological inhibition of RORα also worsened CNV. In addition, both genetic deficiency and inhibition of RORα substantially increased vascular growth in isolated mouse choroidal explants *ex vivo*. RORα inhibition also promoted angiogenic function of human choroidal endothelial cell culture. Together, our results suggest that RORα negatively regulates pathological CNV development in part by modulating angiogenic response of the choroidal endothelium and inflammatory environment in the choroid/RPE complex.

## INTRODUCTION

Choroidal neovascularization (CNV) leads to rapid deterioration of visual function in neovascular age-related macular degeneration (AMD), a common and complex eye disease in the elderly. Abnormal growth of leaky choroidal vessels beneath the retina causes fluid exudation and edema, thereby resulting in retinal detachment and vision loss [1]. While CNV affects only about 10% of AMD patients, it causes up to 90% of vision loss associated with AMD. Both angiogenic and inflammatory factors contribute to CNV, with vascular endothelial growth factor (VEGF) and its signaling being the most well studied [2–5]. Intraocular injections of anti-VEGF compounds have been successful in treating neovascular (wet) AMD, yet many patients remain unresponsive to these therapies, suggesting additional factors are at work. Development of invasive CNV requires not only elevated VEGF levels [2–5], but also an increased inflammatory state in the eye which is associated with invasion of inflammatory cells [6–8]. Many inflammatory mediators such as TNFα are also linked with the development of CNV [8–10]. In addition, higher dietary fat intake [11, 12] and impaired lipid transport [13] are implicated in AMD. Both free and oxidized lipid metabolites including cholesterol and ApoB-containing lipoproteins are found in human drusen [14–16], a hallmark of AMD, suggesting a close link between lipid metabolism and AMD.

Retinoic acid receptor-related orphan receptor alpha (RORα) is a lipid-sensing nuclear receptor that can bind cholesterol and other cholesterol-derived oxysterols [17, 18], although whether these are physiological ligands is still under investigation. Genetic variations in RORα are linked with a higher risk of developing neovascular AMD in humans [19–21]. Functioning as a transcription factor, RORα is a critical regulator of many biologic processes including circadian rhythm, eye and cerebellar development, regulation of lipid metabolism and inflammation [22, 23]. It mediates the expression of key enzymes and factors in lipid metabolism [24–26], and is also important for immunity and inflammatory disorders [22, 27–30]. Ligand binding regulates the interaction of RORα with its transcriptional co-activators and/or co-repressors, the balance of which controls its resultant transcriptional activity [22]. Upon binding to a specific ROR response elements (RORE) in the regulatory region of target genes, RORα and its cofactors together mediate the expression of target genes to impact cellular processes [22].

In the retina, RORα has been localized in retinal neurons including retinal ganglion cells (RGC) and photoreceptors [31–33]. Our previous work found that RORα is expressed in both inflammatory cells, including retinal macrophages and microglia, and RGCs and regulates pathological retinal angiogenesis in a mouse model of oxygen-induced retinopathy modeling ischemic proliferative retinopathy [34, 35]. Whether RORα regulates CNV development remains unclear and is the focus of the current study.

Here, we investigated whether RORα regulates CNV using a mouse model of laser-induced CNV, mimicking the neovascular features of wet AMD. We found that expression of RORα was enriched in the mouse choroid/RPE complex and upregulated in laser-induced CNV. In Staggerer mice (*Rora^sg/sg^*) with spontaneous mutation of RORα resulting in loss of its function [36], genetic deficiency of RORα significantly increased the size of laser-induced CNV lesions and associated vascular leakage. Treatment with an inverse agonist of RORα also worsened laser-induced CNV. Both genetic loss and pharmacological inhibition of RORα enhanced vascular expansion in choroidal explants *ex vivo*. Modulation of RORα also directly impacted choroidal vascular endothelium angiogenesis. Mechanistically, we found that loss of RORα led to upregulation of VEGF receptor 2 (VEGFR2) and TNFα in mouse choroidal/ RPE complex under normal conditions and following laser-induced CNV. These observations suggest that RORα may negatively regulate pathological CNV through modulation of both angiogenic and inflammatory pathways.

## MATERIALS AND METHODS

### Animals

All animal studies were approved by the Institutional Animal Care and Use Committee at Boston Children’s Hospital. The studies also adhered to the Association for Research in Vision and Ophthalmology Statement for the Use of Animals in Ophthalmic and Vision Research. Heterozygous mutant *Staggerer* (*Rora^+/sg^*), B6.C3(Cg)-*Rora^sg^*/J, mice (stock no. 002651) were purchased from Jackson Laboratory (Bar Harbor, ME, USA) and bred to produce age-matched wild type (WT) and homozygous mice for this study. In addition, C57BL/6J mice (stock no. 000664, Jackson Lab) were used for agonist treatment experiments.

### Laser-induced CNV

Laser photocoagulation was performed with Micron IV imaging system (Phoenix Research Lab, Pleasanton, CA, USA) as previously described [37, 38]. Briefly, young adult (2–3 months old) male *Rora^sg/sg^* and WT mice were anesthetized. Male mice were used to avoid influence of sex-hormone on biological variations of CNV response in female mice as reported previously [37, 39]. After pupil dilation, each eye received four laser burns spaced evenly around the optic disc. The laser rupture of Bruch’s membrane was confirmed by the presence of a vapor bubble. Lesions with no observation of bubbles and malformed lesions (fused, or with hemorrhage) were excluded from the study based on previously established criteria [37]. Seven (7) days post-laser, CNV was analyzed in choroidal flat mounts with isolectin B_4_ (Invitrogen, I21413) staining to visualize and quantify lesion size. In addition, before euthanizing mice, fundus fluorescein angiography was performed and the severity of CNV lesion leakage was graded [40].

For pharmacological modulation of RORα, injection of RORα inverse agonist (SR3335) and agonist (SR1078) was performed in 6-8-week-old male C57BL/6J mice with daily i.p. injection (b.i.d.) from day 0–7 post-laser, at a dose of 15 mg/kg (body weight) for both compounds [41, 42]. Both compounds were synthesized and provided by coauthors T.M.K. and L.A.S.’s groups at the Scripps [43].

### Fluorescein fundus angiography (FFA)

Fluorescein angiography was performed 7 days after the laser photocoagulation [37]. Photographs were taken with Micron IV imaging system after injection of Fluorescein AK-FLUOR (100 mg/ml, NDC 17478-101-12, Akorn, Lake Forest, IL, USA). AK-FLUOR stock was diluted to 10 mg/mL working solution and injected i.p. at 10 μL/g (mouse body weight). The lesions were graded as described previously [40], on an ordinal scale defined by the spatial and temporal pattern of hyperfluorescence: grade 0 (no leakage); grade 1 (questionable leakage); grade 2A (leaky); grade 2B (pathologically significant leakage).

### Choroidal sprouting assay

Sprouting of isolated choroidal explants was assayed as previously described [38, 44]. Peripheral parts of the choroid & sclera layer isolated from 6-8-week-old mice were cut into small pieces. Choroidal explants were then grown at 37°C with 5% CO_2_ on growth factor-reduced Matrigel (30 μL/well; BD Biosciences, San Jose, CA, USA) in 24-well plates containing CSC complete medium (Cell Systems, Kirkland, WA, USA) with media change every other day. Images of explants were taken 4 days after plating using a ZEISS AxioOberver.Z1 microscope. The area of explant sprouting was quantified with ImageJ using a semi-automated macro plug-in. Treatment with RORα inverse agonist (SR3335) and agonist (SR1078) (5 μM) or DMSO as control were performed in C57BL/6J choroidal explants.

### Human choroidal endothelial cell (hCEC) culture and MTT and migration assays

HCECs were purchased (Celprogen, 36052-03) and cultured in endothelial cell complete medium (M36052-03S, Celprogen) on extra-cellular matrix coated dishes Celprogen) according to vendor instruction. Cells between passage number 4 and 7 were treated with SR3335, SR1078 (both 10 μM) or vehicle DMSO. Cell viability and/or proliferation was assessed after treatment using an MTT (3-(4,5-dimethylthiazol-2-yl)-2,5-diphenyltetrazolium bromide) cell metabolic activity assay kit (V13154, Fisher Scientific) as described previously [45]. Cell migration assay was carried out according to previous protocols [46].

### Tissue and cell preparation for real time quantitative polymerase chain reaction (RT-qPCR)

Mouse choroidal sample preparation for RNA includes RPE/choroidal/sclera complex dissected from the eye ball. RPE RNA was isolated and purified from dissected WT eye cups after removal of the retina following previous protocol [47]. Macrophage cells were murine RAW 264.7 cells (TIB-71, ATCC). Human microvascular endothelial cells (hRMEC) were purchased from Cell system (ACBRI 181) and mouse brain smooth muscle cells (mSMC) were from Cell Biologics (C57-6085). Cells were cultured according to vendor instructions respectively.

Total RNA was isolated from the homogenized mouse eye tissues or cells by PureLink^™^ RNA Mini Kit (Invitrogen) according to the manufacturer’s instructions. Synthesis of cDNA was done by reverse transcription with iScript^™^ Reverse Transcriptase (Bio-Rad, Hercules, CA, USA). Quantitative analysis of gene expression was carried out by RT-qPCR using a C1000 Thermal Cycler (Bio-Rad) and the 2X SYBR Green qPCR Master Mix (bimake.com; Houston, TX, USA) with primers for specific genes. Copy number of each target gene cDNA was normalized to the house keeping genes, *Rn18s or Gapdh*, using comparative CT (ΔΔCT) method.

Mouse primers used are listed below:

*Rora*, forward: 5′-TCCCACCTGGAAACCTGCCAGT-3′, reverse: 5′-ATGCGAGCTCCAGCCGAGGT-3′; *Rn18s*: forward: 5′-CACGGACAGGATTGACAGATT-3′, reverse: 5′-GCCAGAGTCTCGTTCGTTATC-3′; *Gapdh*: forward: 5′-AACAGCAACTCCCACTCTTC-3′, reverse: 5′-CCTGTTGCTGTAGCCGTATT-3′.

Inflammatory genes: *Il-1b*, forward: 5′-TTCAGGCAGGCAGTATCACTC-3′, reverse: 5′-GAAGGTCCACGGGAAAGACAC-3′; *Il-6*, forward: 5′-TAGTCCTTCCTACCCCAATTTCC-3′, reverse: 5′-TTGGTCCTTAGCCACTCCTTC-3′; *Nfkb*1, forward: 5′-GGAGAGTCTGACTCTCCCTGAGAA-3′, reverse: 5′-CGATGGGTTCCGTCTTGGT-3′; *Nlrp3*, forward: 5′-ATTACCCGCCCGAGAAAGG-3′, reverse: 5′-TCGCAGCAAAGATCCACACAG-3′; *Tnfa*, forward: 5′-TCCAGTAGAATCCGCTCTCCT, reverse: 5′-GCCACAAGCAGGAATGAGAAG-3′.

Angiogenesis genes: *Ang1*, forward: 5′-AGCTCCACCTCGGGTCTACC-3′, reverse: 5′-TGGTCACTCTGGATCTCATTGG-3′; *Cxcr4*, forward: 5′-AGCCTGTGGATGGTGGTGTTTC-3′, reverse: 5′-CCTTGCTTGATGACTCCCAAAAG-3′; *Dll4*, forward: 5′-TTCCAGGCAACCTTCTCCGA-3′, reverse: 5′-ACTGCCGCTATTCTTGTCCC-3′; *Flt1*, forward: 5′-GTCACAGATGTGCCGAATGG-3′, reverse: 5′-TGAGCGTGATCAGCTCCAGG-3′; *Fzd4*, forward: 5′-TTCCTTTGTTCGGTTTATGTGCC-3′, reverse: 5′-CTCTCAGGACTGGTTCACAGC-3′; *Kdr* (*Vegfr2*), forward: 5′-TTTGGCA AATACAACCCTTCAGA-3′, reverse: 5′-GCTCCAGT ATCATTTCCAACCA-3′; *Notch1*, forward: 5′-CCCTTGCTCTGCCTAACGC-3′, reverse: 5′-GGAGTCCTGGCATCGTTGG-3′; *Pdgf*, forward: 5′-TGTGCCCATTCGCAGGAAG-3′, reverse: 5′-GAGGTATCTCGTAAATGACCGTC-3′; *Plxnd1*, forward: 5′-GCTGACTGTAGCCTATGGGGA-3′, reverse: 5′-GCCATCTGGTGGGATGTCAT-3′; *Tspan12*, forward: 5′-TGCTTGGATGAGGGACTACC-3′, reverse: 5′-AACGTTCCGAAGTACCATGC-3′; *Vegfa*, forward: 5′-GGAGACTCTTCGAGGAGCACTT-3′, reverse: 5′-GGCGATTTAGCAGCAGATATAAGAA-3′.

Human primers used are listed below:

*RORA*, forward: 5′-ACTCCTGTCCTCGTCAGAAGA-3′, reverse: 5′-CATCCCTACGGCAAGGCATTT-3′; *GAPDH*, forward: 5′-CCCTTCATTGACCTCAACTA CA-3′, reverse: 5′-ATGACAAGCTTCCCGTTCTC-3′.

### Western blot analysis

Choroid/RPE complex was isolated from dissected mouse eyes at 1, 3, 5, and 7 days post-laser photocoagulation. Tissues were lysed in RIPA buffer (Thermo Scientific) with protease inhibitors and phosphatase inhibitors (Sigma-Aldrich). Total protein concentration was determined via a bicinchoninic acid (BCA; Thermo Fisher Scientific, 23227) assay. Equal amounts of protein lysates were then denatured using a 1:10 mixture of 2-mercaptoethanol and 4X Laemmli buffer, followed by heating to 100°C for 5 minutes. After SDS-PAGE separation, proteins were transferred to polyvinylidene fluoride (PVDF) membranes and probed with RORα antibody (Abcam, ab60134), VEGFR2 antibody (R&D Systems, AF644), TNFα antibody (Cell Signaling Technology (CST), 11948), and β-actin antibody (CST, 3700). Secondary antibodies used are: HRP-conjugated mouse IgG, rabbit IgG (GE Healthcare UK Limited, NA9310V and NA934V, respectively) and goat IgG (SouthernBiotech, 6160-05). ECL Chemiluminescent Substrate Reagent Kit (Invitrogen) was used to generate signal for densitometry quantification.

### Retinal cross section and immunohistochemistry

Mouse eyes were enucleated and fixed in 4% paraformaldehyde in PBS at room temperature for 1 hour, followed by embedding in optimal cutting temperature (OCT) compound, and frozen for cryosection. Immunohistochemistry on retinal sections was performed as described in previous protocols [35, 48]. Primary antibodies for RORα (Abcam, ab278099) were used and sections were costained with isolectin B_4_ (Invitrogen, I21413) overnight at 4°C. After washing, the retinas were incubated with secondary antibody (Thermo Fisher, A11034) for 1 hour at room temperature followed by imaging with a fluorescence microscopy (Axio Observer Z1, Carl Zeiss Microscopy).

### Statistical analysis

Quantitative data are presented as means ± SEM (standard error of the mean), with the exception of qPCR results, which are represented as the mean ± SD (standard deviation). Asterisks represent the *P*-value according to two-tailed Student’s *t*-test (2 groups), One-way ANOVA (more than 2 groups), or Two-way ANOVA (two factors, more than 2 groups): ^*^P ≤ 0.05; ^**^P ≤ 0.01; ^***^P ≤ 0.001; ^****^P ≤ 0.0001.

### Data and materials availability

The paper contains all methods and data needed to evaluate the conclusions. Additional related data and materials are available upon request.

## RESULTS

### RORα was enriched in the mouse choroid and regulated expression of angiogenic and inflammatory genes

**Figure 1:**
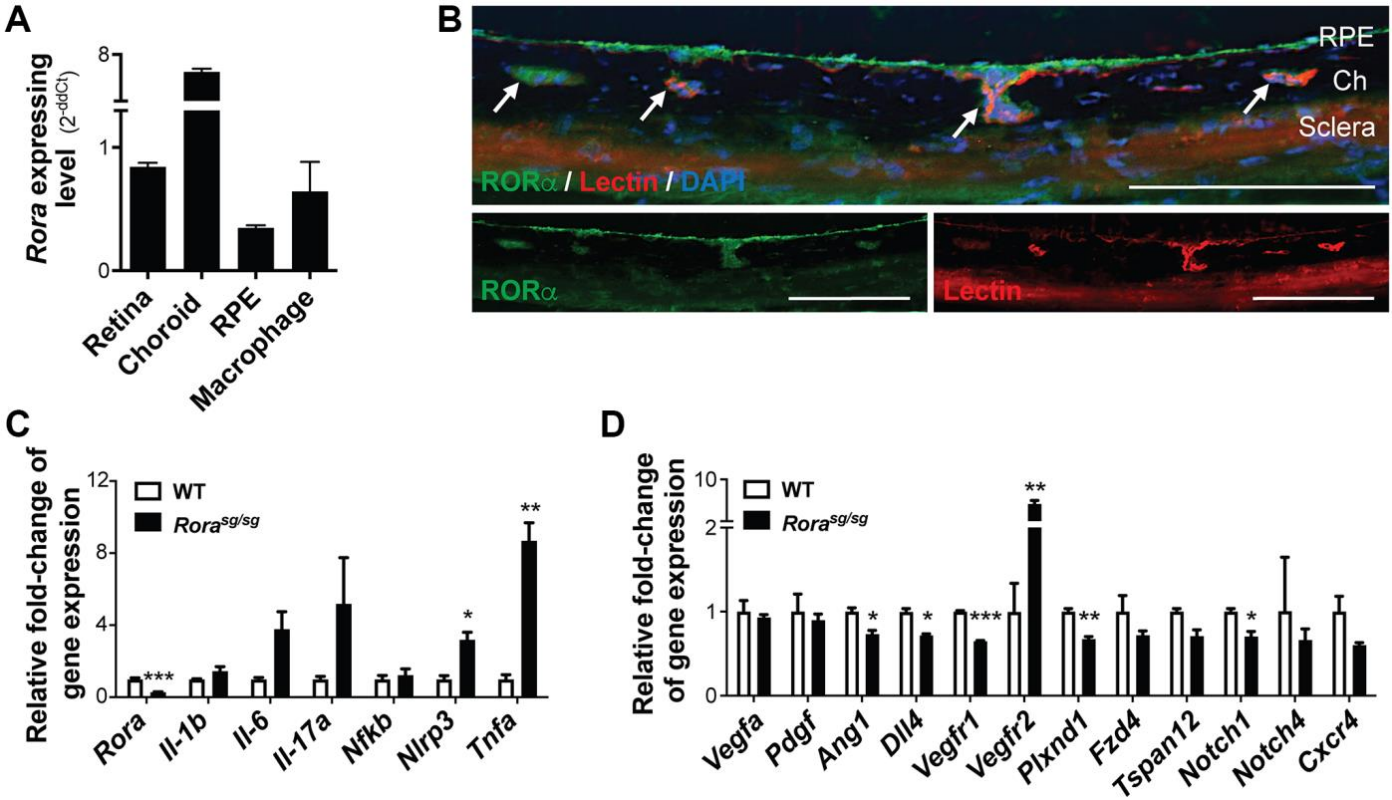
**RORα was enriched in mouse choroid and regulated inflammatory and angiogenic genes.** (**A**) Relative *Rora* expression in different types of mouse ocular tissues, namely retina and RPE/choroid complex, and cells (isolated pure RPE from mouse eyes and RAW264.7 macrophage cell line) measured with quantitative RT-PCR and normalized to housekeeping gene *Rn18s*. The choroid complexes expressed the highest expression levels of *Rora* compared to the retinas, RPE, and macrophage cells (*n* = 3/group). (**B**) Immunohistochemistry staining of retinal cross sections shows RORα antibody staining (green), vascular endothelium marker isolection (red), and DAPI (blue). Ch: choroid. Scale bars, 100 μm. (**C**, **D**) q-PCR analysis for the expression of *Rora* and inflammatory (**C**) and angiogenic (**D**) genes in the RPE/choroid complexes from *Rora^sg/sg^* and WT mice in normal condition without CNV showed that deficiency of RORα led to significant increase in *Vegfr2* and *Tnfa* mRNA levels, in addition to changes in other inflammatory and angiogenic genes (*n* = 3 mice/group). Data are presented as means ± SEM. ^*^P ≤ 0.05; ^**^P ≤ 0.01; ^***^P ≤ 0.001.

We first compared relative gene expression levels of *Rora* in different mouse ocular tissues and cells. Expression of *Rora* mRNA was highly enriched in the normal choroid/RPE complex with about 6-fold increase compared with the retina (Figure 1A). Because the choroid/RPE complex also contains RPE and microglia/macrophages, we also compared *Rora* expression levels in isolated mouse RPE cells and cultured mouse macrophage (RAW264.7) cells, both of which showed lower expression levels than the combined choroid/RPE complex, suggesting that *Rora* is enriched in the mouse choroid (Figure 1A). Immunohistochemistry staining of eye cross sections also showed colocalization of RORα antibody staining with isolectin-positive choroidal vessels, in addition to RORα antibody staining in RPE (Figure 1B).

Next, we evaluated expression of inflammatory and angiogenic genes in RORα-deficient (*Rora^sg/sg^*) choroid/RPE complex. Expression of *Rora* mRNA was very low and barely detectable in *Rora^sg/sg^* choroid/RPE compared with age-matched wild type (WT) controls (Figure 1C), confirming its genetic deficiency. Importantly, *Rora^sg/sg^* choroid/RPE complex had much higher expression levels of inflammatory cytokines, with ~8-fold upregulation of *Tnfa* mRNA, in addition to upregulation of *Il1b*, *Il6*, *Il17a*, and *Nlrp3,* compared with WT (Figure 1C). In addition, expression of VEGF receptor 2 (*Vegfr2*, or *Kdr*) was significantly higher with ~7-fold upregulation in *Rora^sg/sg^* choroid/RPE complex (Figure 1D), while many other angiogenic-related factors (*Vegfa*, *Pdgf*, *Ang1*, and *Dll4*) and receptors (*Vegfr1*, *Plxnd1*, *Fzd4*, *Tspan12*, *Notch1&4*, and *Cxcr4*) were either unchanged or modestly down-regulated. These results indicate that RORα may regulate expression of both angiogenic and inflammatory genes and loss of RORα may promote an inflammatory and angiogenic environment around the choroid.

### Genetic deficiency of RORα worsened laser-induced CNV

**Figure 2:**
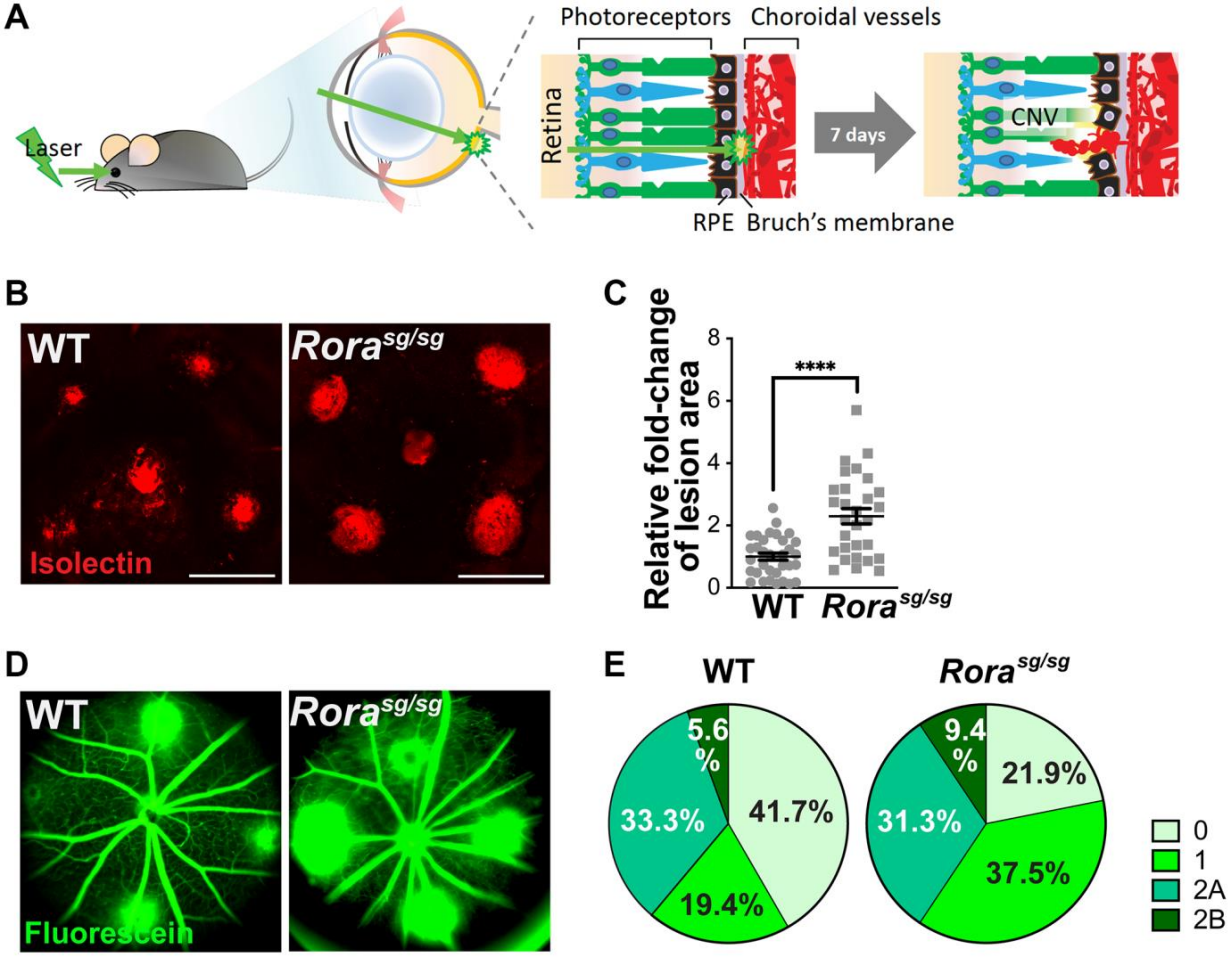
**Genetic deficiency of RORα increased lesion size and vascular leakage in a mouse model of laser-induced choroidal neovascularization (CNV).** (**A**) A cartoon illustrating laser-induced CNV model in mice. Young adult mice are exposed to laser, which ruptures Bruch’s membrane and causes CNV. (**B**) Representative images of choroidal flat mounts with laser-induced CNV from wild type (WT) and RORα-deficient (*Rora^sg/sg^*) mice stained with isolectin IB_4_ (red) showing four lesions, with optic disc in the center. Scale bars, 500 μm. (**C**) Quantification of the relative fold-change of CNV lesion areas showed that RORα-deficient mice have larger CNV lesion sizes compared to age-matched WT (*n* = 22–23 eyes/group). Each data point represents averaged lesion size from one eye. Solid horizontal bars indicate means ± SEM; ^****^P ≤ 0.0001. (**D**) Representative images of fundus fluorescein angiography (FFA) from WT and *Rora^sg/sg^* mice with laser-induced CNV at day 6 after laser photocoagulation. (**E**) Lesions were graded on an ordinal scale of the fluorescein (D; green) leakage appearance: grade 0 (no leakage); grade 1 (questionable leakage); grade 2A (leaky); grade 2B (pathologically significant leakage). *Rora^sg/sg^* mice revealed much fewer grade 0 lesions and more grade 1, 2A and 2B lesions compared to WT mice (*n* = 10 mice/group).

To determine the role of RORα in the regulation of CNV, we used a mouse model of laser-induced CNV to mimic the neovascular aspect of AMD (Figure 2A). Young adult (2–3-month-old) *Rora^sg/sg^* and WT mice were exposed to laser-induced CNV model. At one week post laser, *Rora^sg/sg^* choroidal flat mounts showed greater than 2-fold increase in CNV lesion area compared to WT (Figure 2B, 2C). In addition, genetic deficiency of RORα resulted in a higher percentage of leaky CNV lesions (Figure 2D, 2E). Over 78% of *Rora^sg/sg^* CNV lesions were graded as leaky (including 37.5% of grade 1, 31.25% of grade 2A, and 9.38% of grade 2B lesions), while in WT mice the percentage of leaky lesions was approximately 58% (Figure 2D, 2E). These findings of larger, leakier, and hence more severe CNV lesions in RORα-deficient mice suggest a negative regulatory role of RORα in CNV formation.

### Loss of RORα upregulated VEGFR2 and TNFα protein levels in laser-induced CNV

**Figure 3:**
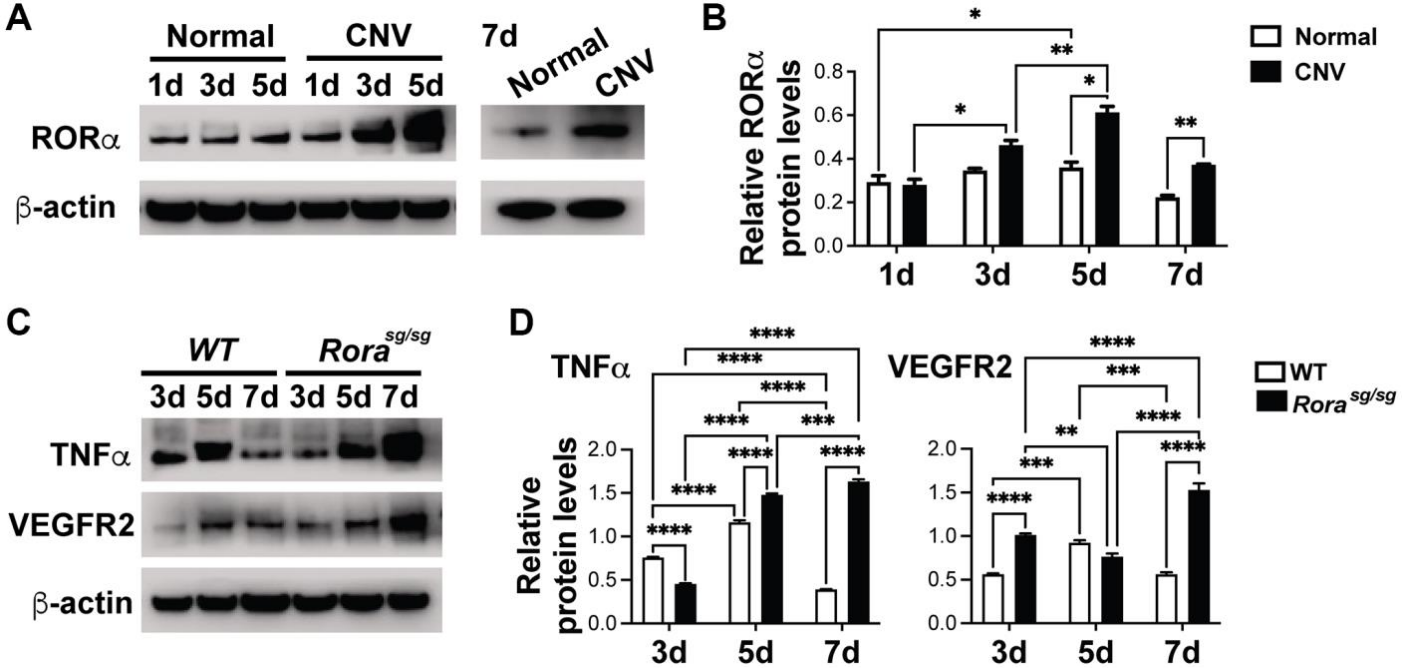
**RORα was induced in CNV and regulated VEGFR2 and TNFα levels in laser-induced CNV.** (**A**, **B**) Western blotting images (**A**) and densitometric analysis (**B**) of protein levels of RORα from choroid/RPE complexes with laser-induced CNV from C57BL/6J mice at 1, 3, 5 and 7 days (d) post-laser, compared with β-actin. β-actin served as loading control. Each band represents pooled sample from 3 retinas. *n* = 3 mice/group. (**C**, **D**) Western blotting images (**C**) and densitometric analysis (**D**) showing TNFα and VEGFR2 protein levels in choroid/RPE complexes at 3, 5, and 7 days after laser-induced CNV in *Rora^sg/sg^* and WT eyes, compared with β-actin as loading control. Each band represents pooled sample from 3 retinas. *n* = 3 mice/group. ^*^P ≤ 0.05; ^**^P ≤ 0.01; ^***^P ≤ 0.001; ^****^P ≤ 0.0001.

Having established that loss of RORα exacerbates laser-induced CNV, we next evaluated whether dysregulation of angiogenic and inflammatory genes such as VEGFR2 and TNFα as seen in normal *Rora^sg/sg^* choroid may stimulate CNV formation. Protein levels of RORα were highly upregulated over time at 1, 3, and 5 days after laser in C57BL/6J mice (Figure 3A, 3B), which may reflect hypoxia-stimulated RORα expression in CNV after laser-induced tissue injury, since RORα is known to be hypoxia-responsive.

Next we evaluated whether VEGFR2 and TNFα, both upregulated in *Rora^sg/sg^* choroid/RPE complex (Figure 1C, 1D) are also affected in *Rora^sg/sg^* eyes with CNV. VEGFR2 is a major receptor for VEGF, the main inducer of both clinical and experimental CNV [5]. On the other hand, TNFα, a major inflammatory cytokine secreted by macrophages, T cells, vascular endothelium and neurons, also primes vascular endothelium for their angiogenic response [49]. We found that protein levels of VEGFR2 and TNFα were strongly upregulated in *Rora^sg/sg^* vs. WT choroid/RPE complex 7 days after laser-induced CNV (Figure 3C, 3D), consistent with worsened CNV lesions in *Rora^sg/sg^* eyes (Figure 2). Together, these findings suggest that genetic loss of RORα may increase laser-induced CNV severity as the result of enhanced VEGFR2 and TNFα levels in the choroid/RPE complex (Figure 3D).

### Genetic loss and pharmacological inhibition of RORα increased choroidal explant sprouting abilities *ex vivo*

**Figure 4:**
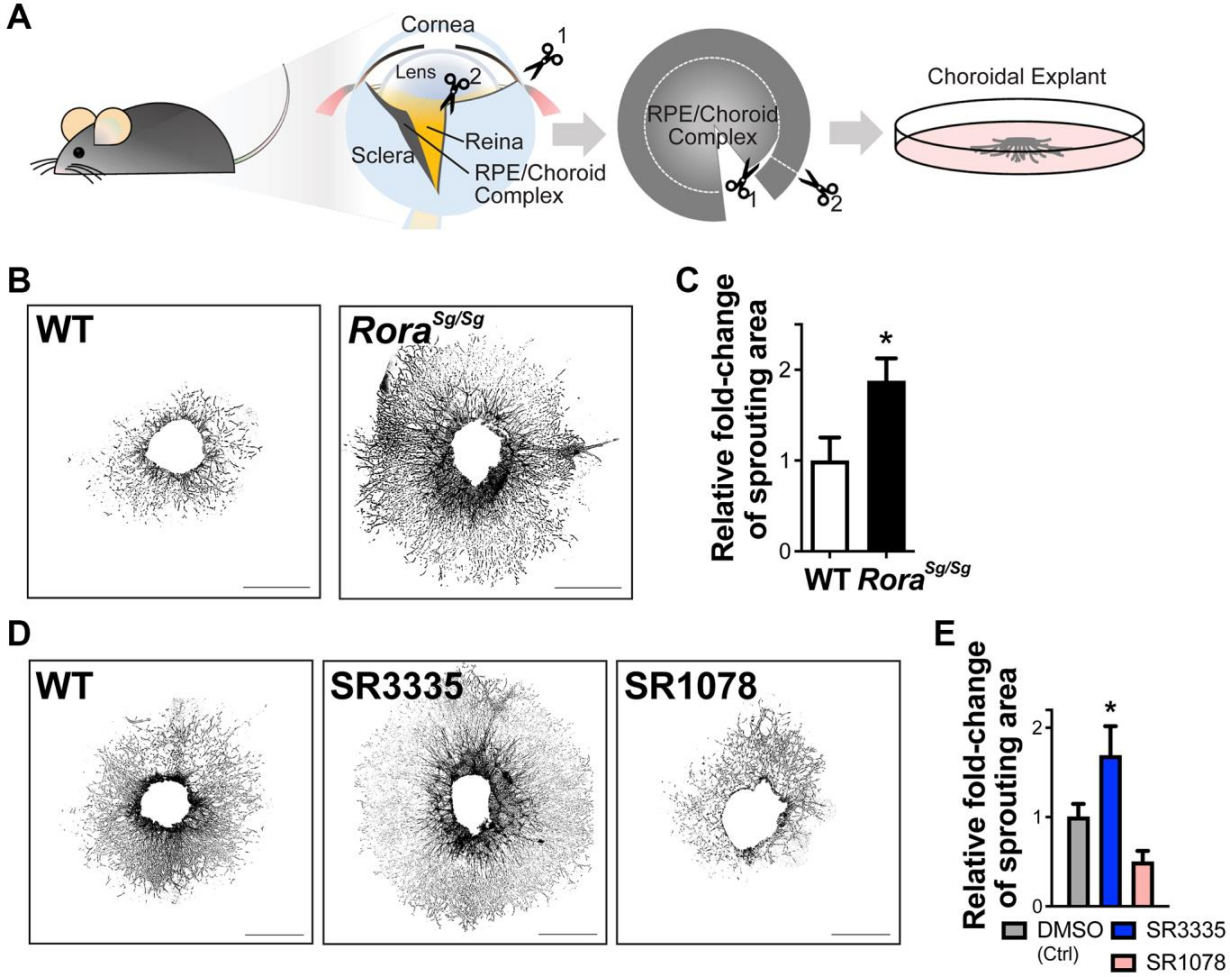
**RORα regulates choroidal sprouting *ex vivo*.** (**A**) A cartoon illustrates the experimental steps of choroidal explant assay by isolation, dissection and culture of choroid fragments. (**B**) Representative images of choroidal sprouting assays from age-matched WT and *Rora^sg/sg^* mice. Scale bars, 1 mm. (**C**) Quantitative analysis of the choroidal sprouting area from 5 days after explantation showed that *Rora^sg/sg^* choroids have significantly increased sprouting ability *ex vivo* compared to WT. *n* = 3–5 mice (10–12 explants)/group. (**D**) Representative images of choroidal explants isolated from C57BL/6J mice and treated with SR3335 (RORα inverse agonist), SR1078 (RORα/γ agonist) or vehicle control DMSO (all at 5 μM). Scale bars, 1 mm. (**E**) Quantification of the sprouting area indicated that inhibition of RORα with SR3335 significantly increased choroidal sprouting area while SR1078 reduced the choroidal sprouting ability compared to the DMSO (control) treated group. *n* = 3 mice/group; 10–12 explants per treatment. Data are presented as mean ± SEM. ^*^P ≤ 0.05.

To explore the effects of RORα on choroidal angiogenic ability, we next performed *ex vivo* sprouting assays using mouse choroidal explants, which partly maintain the cellular matrix and environment in living choroid (Figure 4A). In line with the results from the *in vivo* laser-induced CNV model, choroidal explants isolated from *Rora^sg/sg^* mice exhibited about 2-fold increase in sprouting abilities compared with the choroidal explants from age-matched WT (Figure 4B, 4C), suggesting increased choroidal angiogenic potential in the absence of RORα.

To modulate RORα pharmacologically, synthetic RORα inverse agonist SR3335 [41] and RORα/γ agonist SR1078 [42] were developed. These compounds can bind to the ligand binding domain of RORα to modulate its transcriptional activity [43]. Choroidal explants treated with RORα modulators showed results consistent with *Rora^sg/sg^* mice, where choroidal explants treated with SR3335 to inhibit RORα revealed significantly larger (~60%) sprouting areas compared with the vehicle controls, and RORα activation with SR1078 treatment showed a trend of reduced sprouting ability *ex vivo* (Figure 4D, 4E). Together, these data suggest that both genetic and pharmacological modulation of RORα directly altered the angiogenic and sprouting ability of choroid.

### Pharmacological modulation of RORα affected laser-induced CNV lesions

**Figure 5:**
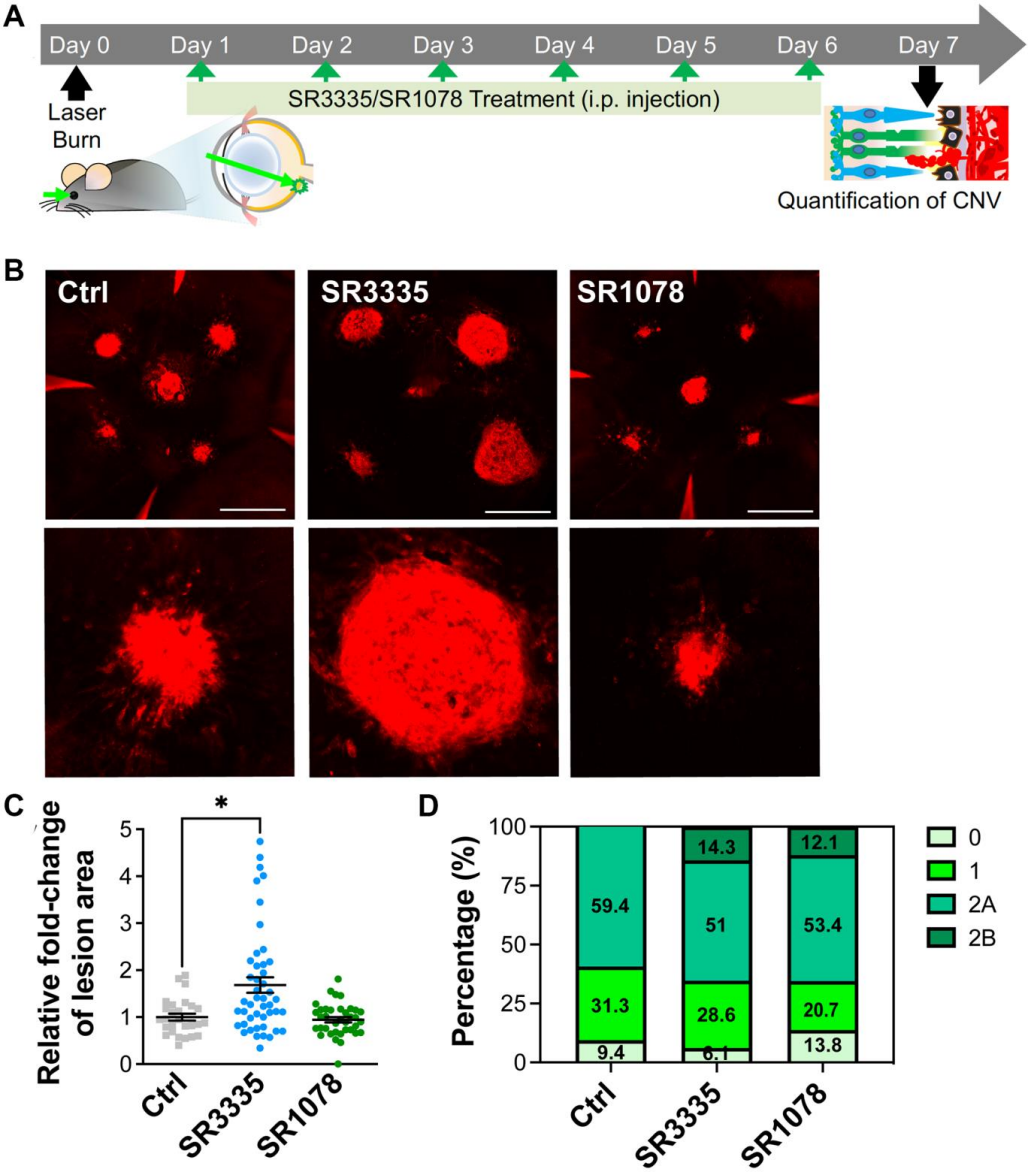
**Pharmacological modulation of RORα affects CNV lesion size in mice.** (**A**) A cartoon of the drug treatment timeline in laser-induced CNV. RORα inverse agonist (SR3335), agonist (SR1078), or vehicle control was intraperitoneally injected (daily) into C57BL/6J mice after laser-induced CNV. (**B**) Representative images of isolectin-stained (red) choroidal flat mounts, isolated from mice of all treatment groups on day 7 after laser photocoagulation. Scale bars, 500 μm. (**C**) Quantification of isolectin-stained CNV area showed significantly increased CNV lesion size in the mice treated with SR3335, and while as the CNV lesion size in the SR1078-treated group did not show significant change, compared to the vehicle control treated group (*n* = 5–8 mice/group). (**D**) Vascular leakage from CNV lesions were assessed by fundus fluorescein angiography (FFA) at day 6 after laser photocoagulation and graded on an ordinal scale of the fluorescein leakage appearance: grade 0 (no leakage); grade 1 (questionable leakage); grade 2A (leaky); grade 2B (pathologically significant leakage). *n* = 5–8 mice/group. Data are presented as mean ± SEM. ^*^P ≤ 0.05.

To further corroborate the effects of RORα on CNV, we administered pharmacological modulators of RORα (inverse agonist SR3335 or agonist SR1078) to C57BL/6J mice (daily intraperitoneal injection) after laser-induced CNV (Figure 5A). SR3335 treatment for RORα inhibition resulted in substantially increased CNV lesion size (Figure 5B, 5C), consistent with our findings in mice with systemic deficiency of RORα (Figure 2). The administration of SR1078 for RORα activation showed a trend of attenuated severity of CNV lesion size (Figure 5B, 5C). In addition, more CNV lesions with SR3335 treatment and less with SR1078 were graded as leaky in grade 1 and 2 (Figure 5D). One might note that the percentage of overall leaky lesion differs in control-treated C57BL/6J mice vs. WT mice (in Figure 2), potentially reflecting difference in mouse colony, FFA procedures and the inherent variability in the CNV model itself. Together these data indicating that pharmacological modulation of RORα activities may influence the development of CNV and could serve as a potential strategy for controlling pathological CNV.

### Angiogenic function of human choroidal endothelial cells was directly regulated by pharmacological modulation of RORα

**Figure 6:**
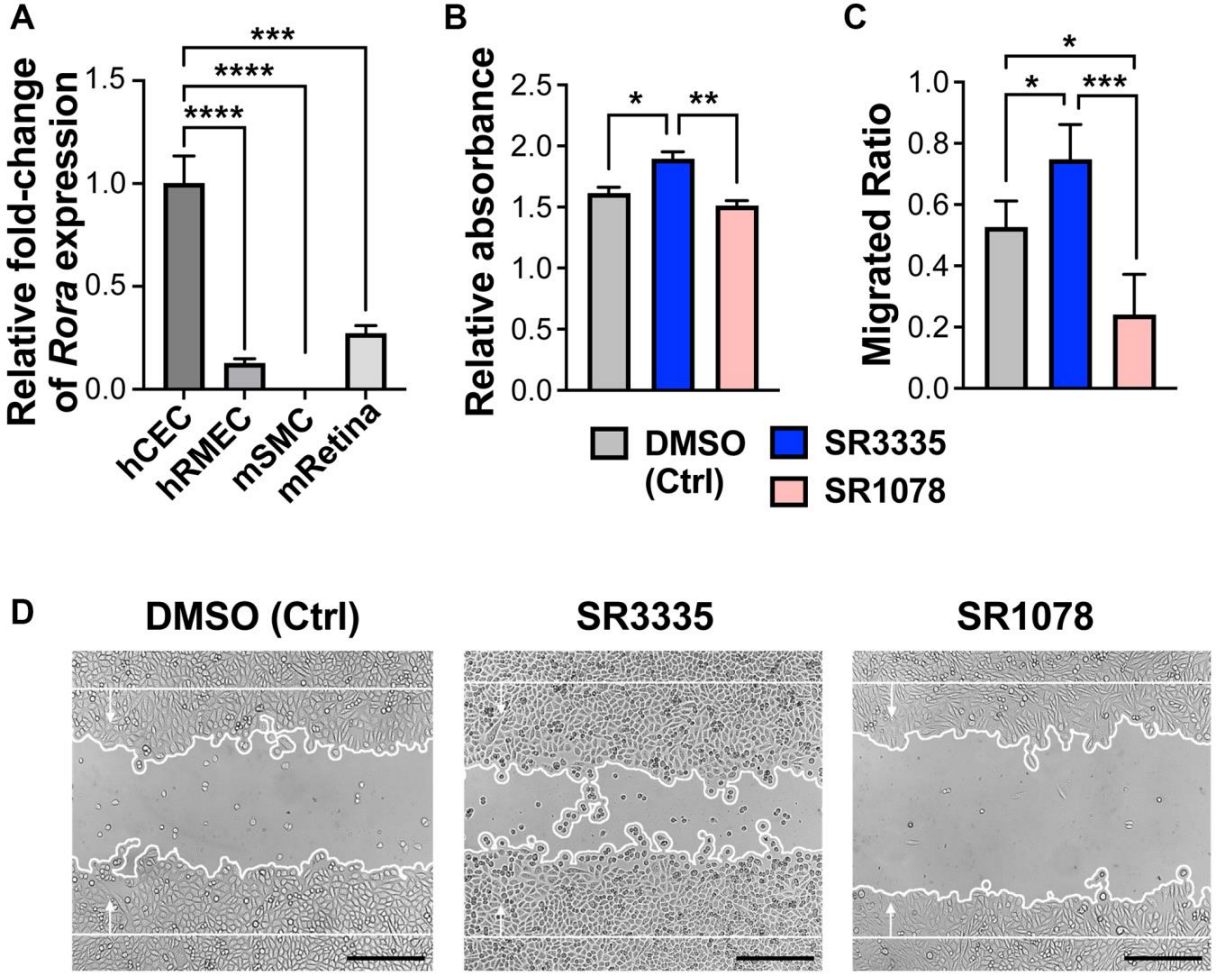
**Pharmacological modulation of RORα regulates human choroidal endothelial cell angiogenic function.** (**A**) Relative mRNA expression of RORα in human choroidal endothelial cell (hCEC), human retinal microvascular endothelial cell (hRMEC), mouse brain smooth muscle cell (mSMC) and mouse whole retina (mRetina), measured with quantitative RT-PCR and normalized to housekeeping gene *GAPDH* (human) and *Gapdh* (mouse) respectively. *n* = 3/group. (**B**) HCECs were treated with RORα inverse agonist (SR3335), agonist (SR1078), or DMSO vehicle control. MTT assay was performed to evaluate cell viability and proliferation. Cell growth was calculated as fold change of relative absorbance normalized to the values at 0 hr. *n* = 3/group. (**C**, **D**) Quantification analysis (**C**) and representative images (**D**) of hCEC migration assay. Cells were grown to confluence and treated with SR3335, SR1078, or DMSO vehicle control. Mitomycin was used to inhibit cell proliferation. A scratch wound was generated in the cells. Cell migration were measured after 24 hr and quantified as new cell coverage areas normalized by the original wound areas. *n* = 4/group. Scale bar: 250 μm. ^*^P ≤ 0.05; ^**^P ≤ 0.01; ^***^P ≤ 0.001; ^****^P ≤ 0.0001.

To evaluate whether RORα may function directly in human choroidal endothelium, we first assessed relative mRNA expression levels of RORα in human choroidal endothelial cells (hCECs), human microvascular endothelial cells (hRMECs), mouse brain smooth muscle cells (mSMCs) and whole mouse retina. Smooth muscle cells were examined because of their presence in choroid vascular tissue. RORα mRNA was highly expressed in hCECs as compared with hRMECs, and expression of RORα mRNA in mSMCs was barely detectable (Figure 6A), confirming enrichment of RORα in choroidal vascular endothelium.

Next, hCECs were treated with RORα inverse agonist SR3335 or agonist SR1078. Choroidal endothelial cell viability and/or proliferation was assessed using MTT assay. We found that SR3335 treatment showed significant increase (*p* ≤ 0.05) in cellular metabolic activity of hCECs as compared to the vehicle control, whereas a decreased trend was observed upon SR1078 treatment (Figure 6B). In addition, RORα inhibition with SR3335 significantly promoted hCEC migration, and in contrast, RORα activation with SR1078 suppressed hCEC migration (Figure 6C, 6D). These data suggest that pharmacological modulation of RORα activities directly regulates choroidal angiogenesis in the vascular endothelium, which underlies the influence of RORα on the development of CNV lesions.

## DISCUSSION

In this study, we present findings for a protective role of RORα in a mouse laser-induced CNV model of neovascular AMD. We found that the expression of RORα was enriched in the mouse choroid and particularly choroidal endothelium, consistent with previous work finding presence of RORα in human aortic vascular endothelium [50]. Both genetic deficiency and pharmacological inhibition of RORα worsened laser-induced CNV, suggesting an anti-angiogenic role of RORα in CNV, in line with a previous study in a hind limb ischemia model that reported increased ischemia-induced angiogenesis in *Rora^sg/sg^* mice [51]. Previously in an oxygen-induced retinopathy model and in *Vldlr^−/−^* mice with spontaneous subretinal neovascularization, we found that either genetic loss or pharmacological inhibition of RORα suppressed retinal neovascularization in neonatal mice [34]. These results together reflect distinct tissue-specific anti-angiogenic roles of RORα in regulating adult tissue angiogenesis in the choroid and peripheral organs, which may differ from its pro-angiogenic role in neonatal retinal vasculature under pathological conditions. Indeed, RORα is expressed in much higher levels in choroidal endothelium than in retinal microvascular endothelium (Figure 6). In addition, RORα deficiency stimulated a pro-inflammatory environment in the CNV choroid in this study, whereas in the oxygen-induced retinopathy model [34], RORα deficiency lead to an anti-inflammatory profile in neonatal retinas. These endothelial specific and inflammatory difference together may underlie in part the different vascular response to RORα in the two ocular vascular beds and angiogenesis models.

**Figure 7:**
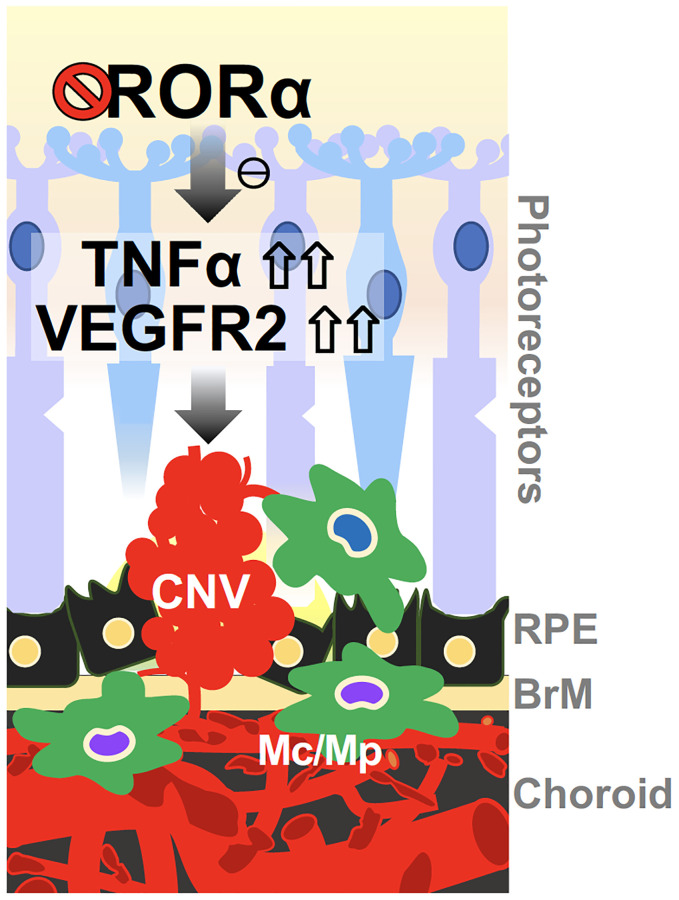
**A schematic model for the effects of RORα on regulating CNV in wet AMD.** Deficiency of RORα in choroid vessels directly induces expression of VEGF receptor VEGFR2, leading to enhanced choroidal endothelial angiogenic response and exacerbated pathological CNV formation. RORα deficiency may also influence CNV via increased TNFα, and chronic inflammation in the choroidal local environment, to potentially sensitize VEGF angiogenic response and thereby CNV formation. Abbreviations: BrM: Bruch’s membrane; CNV: choroidal neovascularization; Mc: microglial cell; Mp: macrophage; RPE: retinal pigment epithelium.

*Rora^sg/sg^* choroid with CNV exhibited enhanced levels of VEGFR2 and TNFα proteins, which may explain the exacerbated laser-induced CNV lesions. VEGFA is the major inducer of CNV in wet AMD and also the most important angiogenic factor in experimental CNV [5]. While loss of RORα did not significantly alter expression of *Vegfa*, it enhanced VEGFR2 expression and thereby VEGF signaling response, which contributes to CNV formation. Other angiogenesis-related genes including VEGFR1 showed modest changes in mRNA levels suggesting their potentially limited impact. VEGFR2 is expressed abundantly in vascular endothelium including the choroid, and RORα expression was also found to be enriched in the choroid, suggesting a vascular specific role RORα of choroidal RORα in regulating CNV. This notion is consistent with our findings in *ex vivo* choroidal explants and hCECs, where genetic loss and pharmacological inhibition of RORα both promoted vascular growth in choroidal explants, and RORα modulation directly regulated hCEC angiogenesis. In addition, TNFα protein levels were also upregulated in *Rora^sg/sg^* choroid, along with several other inflammatory cytokines and factors such as Nlrp3, further promoting the choroidal inflammatory environment to potentially sensitize VEGF response and exacerbate CNV (Figure 7). Previously, inhibition of VEGF or TNFα was found to block or reduce laser-induced CNV in a monkey model [52]. Together our findings suggest that RORα may regulate both VEGF and TNFα to regulate both angiogenesis and inflammation in CNV. Previously in an oxygen-induced retinopathy model we found that genetic deficiency of RORα regulates macrophage polarization and retinal inflammation with dampened TNFα [34]. RORα also plays a critical role in regulating T_H_17-driven inflammatory disorders [27, 30], suggesting a diverse and tissue-dependent role of RORα in inflammation regulation. As a constitutively active transcription factor, it is unclear through what mechanisms loss of RORα induced upregulation of VEGFR2, TNFα and other downstream factors. Whether this reflects direct transcriptional repression through potentially negative response elements [53] on their RORE sites, or indirect regulation via other intermediate factors will await further studies.

We found enriched levels of RORα in the mouse choroid and human choroidal vascular endothelial cell culture, consistent with a recent report of nuclear receptor atlas showing abundant levels of RORα in freshly isolated choroid and primary choroidal endothelial cells from human donors [54]. RORα was also present in RPE and macrophages. Macrophages contribute greatly to formation of CNV [55], whereas RPE, a main producer of secreted VEGF, also influences CNV formation significantly. Therefore, potential cell-specific contribution of RORα from RPE or inflammatory cell sources towards the observed CNV effects cannot be excluded. In addition, localization of RORα was also reported in retinal neurons such as RGCs [33, 35] and cone photoreceptors [56], although the relative contribution of RORα in these retinal neurons in CNV formation is likely limited. Choroid tissue also contains smooth muscle cells, although our analysis of mouse brain smooth muscle cells showed undetectable levels of RORα expression (Figure 6). A previous study found RORα expression in human aortic smooth muscle cells [50], hence RORα expression in smooth muscle cells may be organ- or species-dependent. Additional investigation exploring the cell specific contribution of RORα in CNV will help address this limitation of the current study. Studying other potential molecular targets of RORα in CNV is also needed in future work, as well as the physiological roles of natural RORα ligands in CNV formation.

The natural ligands for RORα are cholesterol derivatives, and RORα regulates cholesterol homeostasis in the liver [57]. Dyslipidemia is closely linked with clinical AMD, and both cholesterol and cholesteryl fatty acid esters are found to be highly concentrated in the extracellular milieu around Bruch's membrane and enriched in drusen [58]. One of them, 7-ketocholesterol, accumulates with age in ocular tissues and in drusen [58] and promotes choroidal endothelial cell migration and neovascularization by inducing endothelial-mesenchymal transition [58]. RORα may be the link between these cholesterol metabolites and choroidal angiogenesis and inflammation, leading to worsened CNV in the absence of RORα.

Pharmacological modulation of RORα with a synthetic agonist and inverse agonist regulated vascular growth in choroidal explants and in laser-induced CNV, suggesting RORα may be a potential druggable target for managing CNV. RORα may target both angiogenic and inflammatory pathways, which can offer more advantage than targeting a single pathway. While SR1078 only showed limited protection towards choroidal explant and CNV in this work, future development of more potent or more efficient RORα-specific agonists may provide better protection. Currently, AAV-delivery of RORα (OCU410) is being evaluated by Ocugen Inc. (Malvern, PA, USA) as a potential dry AMD treatment with a planned clinical trial. Targeting RORα and its related pathway may thus provide a new way to tackle CNV in late neovascular AMD or even early dry AMD by addressing both angiogenic and inflammatory pathogenic factors, which can offer more advantage than targeting a single pathway.
